# Multi-scale non-uniform hierarchical filtering model based on fractal theory

**DOI:** 10.1371/journal.pone.0315423

**Published:** 2025-02-05

**Authors:** Jian Zhang, Fei Peng, Chao He

**Affiliations:** 1 College of Mechanics and Transportation, Southwest Forestry University, Kunming, Yunnan, China; 2 School of Intelligent Manufacturing, Panzhihua University, Panzhihua, Sichuan, China; University of Sharjah, UNITED ARAB EMIRATES

## Abstract

Gasoline particulate filters (GPF) are widely used due to their superior environmental benefits, but its trapping efficiency is affected by many factors. We established a multi-scale non-uniform hierarchical filtering model (MNHF) based on the fractal theory to accurately analyze the dynamic changes of trapping efficiency during GPF operation. The multi-scale characteristics of the filter wall about trap diameter and pore size are presented. Additionally, filter theory and Brownian kinematics are used to precisely predict particle motion. The study focuses on the dynamic change of trapping efficiency of MNHF in different particle size ranges. The results indicate the following: By comparing the numerical simulation results of the model with experimental data, the maximum relative error range is found to be within 0.7%. The MNHF model accurately predicts the change in trapping performance at different times when particles move in the trap. The trapping efficiency of the upper layer of the single-layer trap is higher than that of the lower layer based on the particles’ moving distance in unit time, and the trapping efficiency of the next layer is reduced by up to 29.34% compared to that of the upper layer. Additionally, it provides a more accurate simulation of the trapping efficiency for particles with sizes ranging from 0.01 μm to 0.5 μm under conditions of low wall flow velocity.

## Introduction

Gasoline engines are widely used in vehicles due to their high thermal efficiency, low fuel consumption, and high power. However, some particulate matter emitted from gasoline engines is believed to have carcinogenic effects on humans [1]. In addition to the adverse effects on human health, emissions from gasoline engines can lead to reduced visibility, global warming, building pollution, significant damage to exposed materials, and diminished groundwater quality. Most of the emissions from gasoline engines consist of fine particles. Through electron microscope observations, the particles emitted by gasoline engines can be classified into two categories: nuclear states and condensed states. These particles are a collection of irregular shapes [2–4]. When studying the filtration characteristics of Gasoline particulate filters (GPF), the classical filtration theory based on the assumption that particles are spherical fails to adequately describe the aerodynamic, mass, and dimension characteristics of fractal particles. However, by incorporating fractal theory into the classical filtration theory, the trapping performance of fractal particles can be effectively simulated [5–8].

To improve trapping efficiency, researchers have explored optimizing the structural parameters of the trap. Saito et al. [9] studied the effect of average pore size and wall thickness on trapping efficiency and found that reducing the pore size and increasing the wall thickness improved trapping efficiency. Moreover, as the volume of the filter increased, the airflow velocity decreased, leading to particle accumulation and reduced porosity, thus improving trapping efficiency. Considering the effect of porosity and pore size on pressure drop, Ito et al. [10] concluded that increasing porosity tended to decrease pressure drop. While studying the influence of wall thickness and whole density on trapping efficiency, it was found that changes in thickness had a greater impact than whole density. Lambert et al. [7] studied the influence of filtration materials on trapping efficiency through porosity measurement and cold flow experiments. They determined that even small changes in pore size and porosity could still decrease permeability through cold flow experiments. When permeability dropped below 0.3 μm2, the influence on pressure drop became more significant. In the pursuit of an optimal filter wall design, Joshi et al. [11] studied porosity and pore size distribution and suggested that a tight pore size distribution and porosity in the range of 0.45–0.5 could improve trapping efficiency. While studying filter structure parameters is beneficial for understanding trapping performance during the actual trapping process of GPFs, it cannot accurately predict the change in trapping performance during the subsequent GPF trapping process.

Researchers Yang et al. established a mathematical model to dynamically analyze the trapping efficiency during the trapping process based on the study of the influence of structural parameters. They employed a pore filtration model, taking into account the differences between diesel particulate filters (DPFs) and GPFs in particulate matter, exhaust, design, and operation. To simulate the deep-bed filtration process of GPF, they explored various geometric parameters to study the changes in trapping efficiency and pressure drop. However, during the prediction of dynamic changes in pressure drop during the trapping process, a deviation from the late experimental data was observed [12]. Gong and Utland built a heterogeneous multi-scale filtration model (HMF) based on the probability density function and classical filtration theory to calculate the trapping efficiency of a clean particulate filter. The HMF model effectively overcame the limitations of the classical average filtration model and emphasized the analysis of uneven porosity and pore size distribution in the filtration model to better represent the filtration characteristics in the dynamic collection process [8, 13]. Bonarens et al. [14] proposed that multi-dispersed filling structures could more accurately express the heterogeneity of the microstructure. Liu et al. [15] established a statistical capillary model to simulate the trapping efficiency and permeability during the trapping process. By considering porosity, pore size distribution, and curvature as structural parameters, they verified the prediction accuracy of the model by analyzing the filter’s permeability and trapping efficiency, establishing correlations between the filtration path and filtration performance. Walter et al. used 3D X-ray microscopy to observe the dynamic changes in the microstructure of gasoline engine particulate traps under driving conditions. They validated and extended some filtration models by simulating real driving conditions, leading to more accurate predictions of the trapping process [16, 17]. Despite their dynamic analysis of predicted trapping efficiency, the above models required high adjustments to trapping and structure parameters and didn’t consider the fractal characteristics of particles and trapping channels. Consequently, the simulated efficiency deviated from the actual efficiency data. While these models successfully predict the dynamic changes in trapping performance during the deep-bed filtration stage, they cannot accurately simulate the subsequent stage’s trapping performance.

In this paper, a multi-scale non-uniform hierarchical model (MNHF) based on fractal theory is established to predict the dynamic changes in the trapping efficiency of the particulate trap during the trapping process. The MNHF model considers the non-uniform distribution characteristics of the pore size and incorporates the fractal characteristics of the particles and the trap channel. The application of the MNHF model is verified using the experimental data and analyzing the structural parameters and conditions. By accounting for the non-uniformity of the particles’ moving distance at different times, the MNHF model more accurately reflects the dynamic changes in the trapping performance of the trap.

## Model building

### Particle distribution

The dynamic change of porosity during trapping is related to the deposition mass on the surface of the trap, and the calculation of deposition mass is dependent on the particle size. Particle mass can be determined by analyzing its mass distribution. Particle size distribution refers to the proportion of different particle sizes in a particle swarm. Under certain conditions, mass distribution and particle size distribution represent the mass proportion. It is assumed that the particle mass is positively correlated with the third power of the particle size for all particles at the same density. The mass frequency of particles in the i-th particle size range can be obtained as follows:

$$g_{i}=\frac{m_{i}}{\sum m_{i}}=\frac{n_{i}d_{pi}^{3}}{\sum_{1}^{n}d_{pi}^{3}}$$ (1)

The expression of cumulative frequency under the mass screen is:

$$G_{i}=\sum_{1}^{n}g_{i}=\frac{\sum_{1}^{i}d_{pi}^{3}}{\sum_{1}^{N}d_{pi}^{3}}$$ (2)

The mass frequency density is:

$$Di=\frac{dG}{dd_{p}}$$ (3)

where m is particle mass, *d*_*p*_ is particle size. The mass distribution of these particles can be obtained by reference [18].

### Dynamic change of porosity

By using the pressure pump method to measure the pore size distribution, a distribution curve can be obtained [19]. The study finds that the pore size distribution can be fitted with a lognormal distribution, and the characteristic parameters are average pore size and variance [20]. The probability density distribution *PDF*_*dpore*_ of the pore size is known, and the probability density distribution ${PDF}_{d_{c}}$ (Eq (5)) of the trap diameter can be derived from the relationship between the trap diameter *d*_*c*_ and the pore size *d*_*proe*_ (Eq (4))

$$d_{c}=\frac{3}{2}\frac{1-\varepsilon }{\varepsilon }d_{proe}$$ (4)

$${PDF}_{d_{c}}(d_{c})=\frac{3}{2}\frac{1-\varepsilon }{\varepsilon }{PDF}_{d_{proe}}$$ (5)

where ε represents porosity.

As the particles pass through, some of them will be deposited on the filter wall. The proportion of particles deposited on the wall to the total particles is characterized by a partition coefficient, which is defined as [21]:

$$\Phi_{t}=\frac{d_{c,t}^{2}-d_{c0}^{2}}{{\left(\psi b\right)}^{2}-d_{c0}^{2}}$$ (6)

where *d*_*c*,*t*_ represents the catcher’s collective diameter at the time *t* of the first discrete layer; *d*_*c*0_ is the diameter of the catcher in a clean state; *ψ*(0<*ψ*<1) is a dimensionless penetration factor, which indicates the closeness between the diameter of the trap and that of the trapping unit, determining the maximum size that the trap can achieve. It is taken as 0.95 in this paper [22]. *b* is trapping unit diameter:

b=dc(1−ε)13 (7)

The filter wall is divided into *N* layers along the direction of wall thickness. It is assumed that the particle mass entering the first layer is:

$$m_{in,1,t}=(1-\Phi_{t})m_{up}$$ (8)

The particle mass trapped at time *t* on each layer is:

$$m_{j,t}=m_{in,j,t}\eta$$ (9)

where, *m*_*in*,*j*,*t*_ is particles entering the layer at the moment *t*, expressed as:

$$m_{in,j,t}=m_{in,j-1,t}-m_{j-1,t}$$ (10)

In the process of trapping, the deposition of particles continuously increases, changing the basic parameters of the trap and affecting the trapping efficiency. Suppose that particles are uniformly deposited on the surface of the trap, causing it to become spherical and grow larger [22]:

$$d_{ci,j,t}={\left[\frac{3}{4\pi }\frac{m_{ci,j,t-1}}{\rho_{s}}+{\left(\frac{d_{ci,j,t-1}}{2}\right)}^{3}\right]}^{\frac{1}{3}}$$ (11)

$$m_{ci,j,t-1}=m_{j,t-1}\cdot {PDF}_{d_{ci,j,t-1}}$$ (12)

where, *m*_*j*,*t*?1_ represents the instantaneous mass of carbon fume trapped by the j-th layer of the trap at the moment *t*−1;*ρ*_*S*_ is the instantaneous mass of carbon fume trapped by the micro-equation trapped by the filter layer.

As the mass of the trapped particles increases, the diameter of the trap changes, resulting in a new trap diameter probability density distribution function. This results in the rule of porosity changing with time being obtained [22].


$$\varepsilon_{t}=1-\frac{\int {d_{ci,t}}^{3}{PDF}_{d_{ci,t}}d(d_{ci,t})}{\int {d_{ci,0}}^{3}{PDF}_{d_{ci,0}}d(d_{ci,0})}(1-\varepsilon_{0})$$ (13)

### Modification of the single-fiber theoretical model

Classical filtration theory neglects to consider the fractal characteristics of fractal particles when studying different filtration mechanisms. To achieve a more accurate representation of the real filtration process in simulated GPF trapping, different equivalent radii are used to correspond to various filtration mechanisms. The specific correction analysis process is as follows:

The theories of diffusion mechanism, direct interception mechanism, and inertial mechanism stem from Aerosol Measurement: Principles, Techniques, and Applications [23]. After an in-depth study of the classical filtration theory by Balazy and Podgorski, corrections are made to account for the characteristics of fractal particles in the filtration process, such as diffusion coefficient, interception coefficient, and Stokes number [24]. The corrected diffusion coefficient expression is as follows:

$$D=\frac{k_{B}TC_{c}}{6\pi \mu_{g}R_{p\cdot mob}}$$ (14)

The expression of the interception coefficient of fractal particles is as follows:

$$N_{R}=\frac{R_{p.out}}{R_{f}}$$ (15)

Stokes number expression of fractal particles is:

$$Stk=\frac{R_{P\cdot mas}^{3}U_{0}\rho_{p}C_{c}}{9\mu_{g}R_{f}R_{p\cdot mob}}$$ (16)

where *R*_*P*.*mob*_ is the equivalent migration radius of fractal polymerization, collectively referred to as migration radius; *R*_*P*.*out*_ is shell’s equivalent radius of a fractal polymer; *R*_*P*.*mas*_ is the mass’s equivalent radius of a fractal polymer; *k*_*B*_ is the Boltzmann constant; *Cc* is the Cunningham slip correction coefficient; *R*_*p*_ is particle radius; *T* is absolute temperature; *μ*_*g*_ is aerodynamic viscosity; *R*_*f*_ is the radius of the trap; *U*_0_ is airflow velocity; *ρ*_*p*_ is particle density.

The relationship between the equivalent migration radius and the rotation radius can be expressed as a function of the fractal dimension, the Craterson coefficient and the primary particle size:

$$\frac{R_{p.mob}}{R_{p.gyr}}=f(K_{n},D_{f},a)$$ (17)

where: *R*_*p*.*gyr*_ is rotation radius of a fractal polymer; *K*_*n*_ is the number of the particles; *D*_*f*_ is particulate fractal dimension; *a* is the radius of the primary particles that make up the fractal particles. The calculation of parameter relations including shell’s equivalent radius, equivalent migration radius, equivalent mass radius and fractal dimension is detailed in references [25, 26], which will not be introduced in detail here.

After determining the diffusion coefficient, interception coefficient, and Stokes number of particles from the above equations, we can express the diffusion mechanism, direct interception mechanism, and inertia mechanism as follows.

Diffusion mechanism [23]:

$$E_{D}={\left(\frac{1-\alpha }{k_{u}}\right)}^{\frac{1}{3}}{Pe}^{-\frac{2}{3}}$$ (18)

Direct interception mechanism [23]:

$$E_{R}=\frac{\left(1-\alpha \right)}{k_{u}}\frac{N_{R}^{2}}{{\left(1+N_{R}\right)}^{m}}$$ (19)

Inertial mechanism [27]:

$$E_{I}=\frac{{Stk}^{2}}{Stk^{3}+0.77Stk^{2}+0.22}$$ (20)

where, *P*_*e*_ = (2*R*_*f*_*U*_0_)/*D* is the Peclet number; *α* is the filter filling density: *m* = 2/[3(1−*α*)].

Single-fiber theoretical overall efficiency (Kulkarni et al. 2011):

$$E=1-(1-E_{D})(1-E_{R})(1-E_{I})$$ (21)

**Fig 1:**
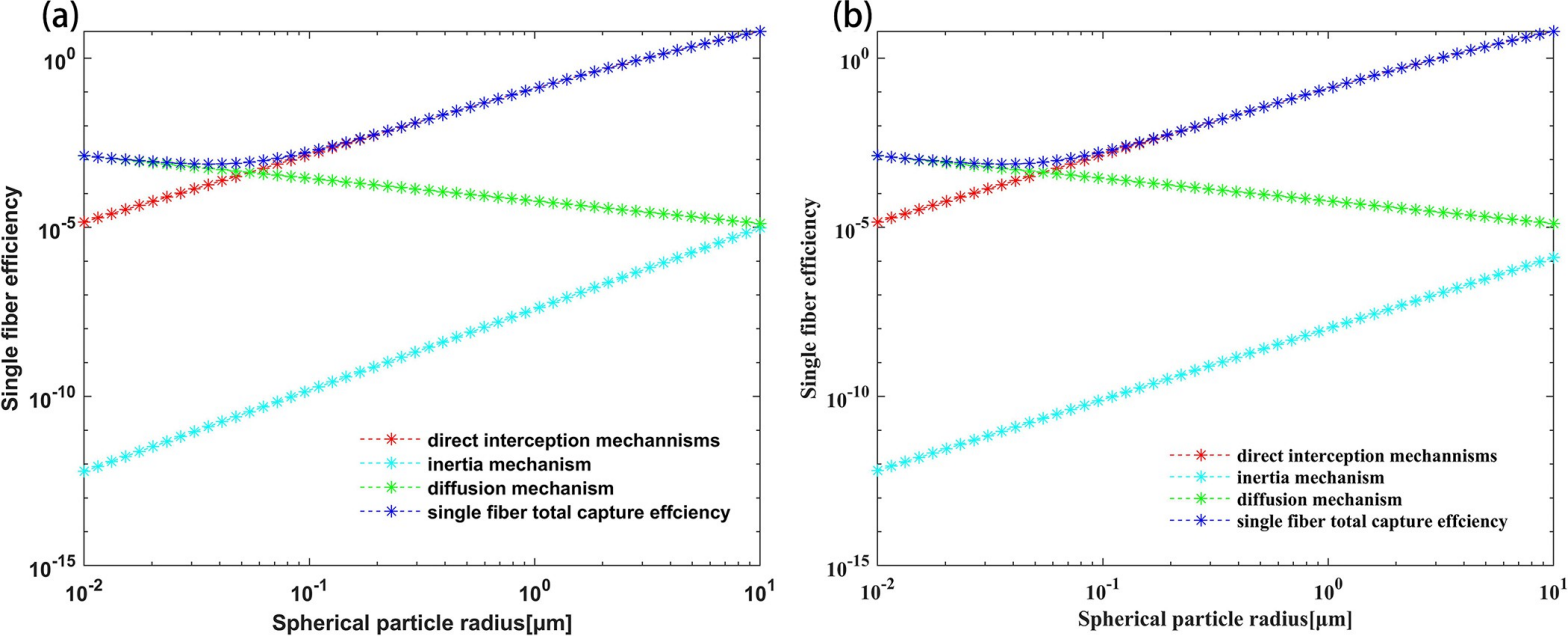
Variation of single-fiber trapping efficiency with particles of different sizes. (a) Fractal aggregate particle with D_f_ = 1.8 and a = 15 nm; (b) Fractal aggregate particle with D_f_ = 1.7 and a = 15 nm.

Fig 1 shows the variation of single-fiber trapping efficiency with particle size for different particles. According to the data [28, 29] the fractal dimension of particulate matter emitted from a gasoline engine ranges from 1.36 to 2.38. The calculation parameters are as follows: the surface flow rate is 1 m/s, the fiber diameter is 20 μm, the filter filling rate is 0.45, the temperature is 293 K, the pressure is 101 KPa, the fractal dimensions of particles are 1.8 and 1.7, and the primary particle size is 30 nm.

As shown in Fig 1, when the particle size is less than 0.1 μm, the trapping efficiency mainly considers the influence of the diffusion mechanism. On the other hand, when the particle size range is greater than 0.1 μm, the direct interception mechanism is considered the primary influence mechanism of particles. Analyzing Fig 1(A) and 1(B), it can be analyzed that when the fractal dimension of particles is lower, their shape becomes more regular, and the surface roughness decreases. This reduces the deposition and adhesion ability of particles in the filter, resulting in a weakened influence of the inertial mechanism on the overall single-fiber trapping efficiency. Consequently, the motion trajectory of particles changes. Li et al. [19], using the same research methods and analyzing Balazy’s simulation conclusions on the filtration mechanism of fiber materials for fractal particles by applying the Brownian dynamic method, drew similar conclusions.

## Brownian dynamics approach

Another method to calculate the efficiency of a single fiber is the Brown Dynamics (BD) method, which avoids the artificial and physical separation of individual mechanisms. Unlike the classical single-fiber theory, it considers the simultaneous action of all mechanisms. The BD method can be used to obtain the deposition efficiency of aerosol particles by tracking the random trajectories of particles and determining whether a given particle collides with others.

For Brownian motion, the motion of a rigid spherical particle with coordinates *q*, mass *m*, and momentum *p* can be described by the Brownian equation of motion [30, 31]:

$$\frac{dq}{dt}=\frac{p}{m}$$ (22)

$$\frac{dp}{dt}=f-\nabla U(q)+\xi (t)$$ (23)

where *t* is time; $f=-\frac{stk}{m}p$ is the frictional resistance of particles; *stk* is Stokes coefficient, and *stk* = 6*πea*, in which *e* is fluid viscosity and *a* is particle radius; *ξ*(*t*) = *λR*(*t*) is the random force generated by particle collision with fluid molecules; $\lambda ={\left(2D_{T}\right)}^{\frac{1}{2}}a$ is the damping coefficient, and $D_{T}=\frac{k_{B}T}{6\pi \eta a}$ is the linear diffusion coefficient; *k*_*B*_ is Boltzmann constant; *T* is the absolute temperature of the system; *R* is Gaussian random noise; ∇*U*(*q*) is the forces on a particle; *U* is the interaction potential between the particles.

For the change of particle velocity, the expected value of particle velocity change *V*1 and the linear displacement of the particle *L*1 on the time step *t* can be expressed as [32]:

$$V1=[u_{i}-V_{i}-\nabla U(q)/\left(MP\right)][1-exp(-P\Delta t)]$$ (24)

$$L1=[u_{i}-\nabla U(q)/\left(MP\right)]\Delta t-[1-\exp\left(-P\Delta t\right)]\times [u_{i}-V_{i}-\nabla U(q)/\left(MP\right)]/P$$ (25)

$$V2=\sqrt{\langle {\Delta v}_{i}^{2}\rangle }=\sqrt{\left(1-e^{-2P\Delta t}k_{B}T\right)/M}$$ (26)

$$L2=\sqrt{\langle \Delta L_{i}^{2}\rangle }=\sqrt{(2P\Delta t-3+4e^{-P\Delta t}-e^{-2P\Delta t})k_{B}T/\left({MP}^{2}\right)}$$ (27)

The expected value of particle velocity change *V*1 be expressed as:

$$u_{i}=\frac{d\psi (t)}{dt}*a$$ (28)

*V*2 is the standard deviation, and *P* is defined as *P* = *f*/*M*.

The correlation coefficient *PI* is given by the following formula:

$$PI=\sqrt{\langle \Delta L_{i}\Delta v_{i}\rangle }={\left(1-e^{-P\Delta t}\right)}^{2}{\left[(1-e^{-2P\Delta t})\times (2P\Delta t-3+4e^{-P\Delta t}-e^{-2P\Delta t})\right]}^{-\frac{1}{2}}$$ (29)

Combining the theory of Brownian motion and single fiber, we start by knowing the particle velocity component and its location. Firstly, we calculate the local flow velocity, the force, *F*^(*ext*)^ and the coefficient of *P*. Then, we determine the expected values *V*2 and *L*2, as well as the correlation coefficients *PI*. Next, two uncorrelated random numbers, $G_{v_{i}}$ and $G_{L_{i}}$ are obtained from Gaussian distributions with zero mean and unit variance. Finally, using the expressions for deterministic and random motion, we calculate the changes in particle velocity and linear displacement at time *t* [24]:

$$V3=V1+G_{v_{i}}V2$$ (30)

$$L3=L1+(PI\sigma_{L_{I}}/B)(V3-V1)+\sqrt{1-PI^{2}}G_{L_{i}}L2$$ (31)

where *L*3 is the linear displacement of the particle, and *L*1 is the linear displacement of the particle.

The final result is:

$$v_{i}(t+\Delta t)=v_{i}(t)+V3$$ (32)

$$L_{i}=\left(L_{i}(t)+L_{3}\right)/\left(t+\Delta i\right)$$ (33)

where, *L*_*i*_ = (*L*_*i*_(*t*)+*L*3)/(*t*+*Δi*) is what we need for the particle moving distance as a function of time.

Obtaining a high trapping efficiency does not guarantee overall effectiveness. We also need to consider the pressure drops, which can be obtained through the equation once the total efficiency of a single filter is determined. The filter trapping efficiency *η* (measured experimentally by the number of particles collected for the filter and the particles entering the filter) can be calculated according to the classical filtration theory as follows:

$$\eta =1-exp(\frac{-2\alpha [1-(1-E_{D})(1-E_{R})(1-E_{I})]L}{\pi R_{f}(1-\alpha )})$$ (34)

where *L* is the thickness of the filter media.

In summary, Eqs (18), (19), (20), (21) and (34) together form the filtration efficiency model. The model can reflect the effects of porosity, flow velocity, collective radius, wall thickness and particle fractal dimension on the trapping efficiency.

Darcy’s Law is an important formula for describing the pressure drop in porous media.


$$\Delta P=\frac{1}{k^{*}}\eta UL$$ (35)

According to Davis’s conclusion [33], the permeability of the medium K* can be described as follows:

K*=Rf216a32[1+56a3] (36)

## Dynamic trapping process

The results obtained using the Brownian dynamics method are compared with those calculated using classical filtration theory, and significant differences are observed in the most penetrating particles. Both methods can reflect the limit case of small or large particles [18].

In Balazy’s conclusion on the BD method [24], it is proven that when the particle radius is less than 1 μm, the trapping efficiency calculated by the single-fiber theory almost coincides with the trapping efficiency obtained by the Brownian motion method. The result of the Brownian motion method shows that the distance the particles travel is decreasing.

In this case, the particles enter the GPF process as follows: when the particles enter the trap from the mouth of the filter, they are affected by the airflow in the orifice and various mechanisms, resulting in the movement curve of the particles in the trap appearing as an irregular folding line. Finally, their motion gradually slows down until they come to a stop and are trapped by the filter.

**Fig 2:**
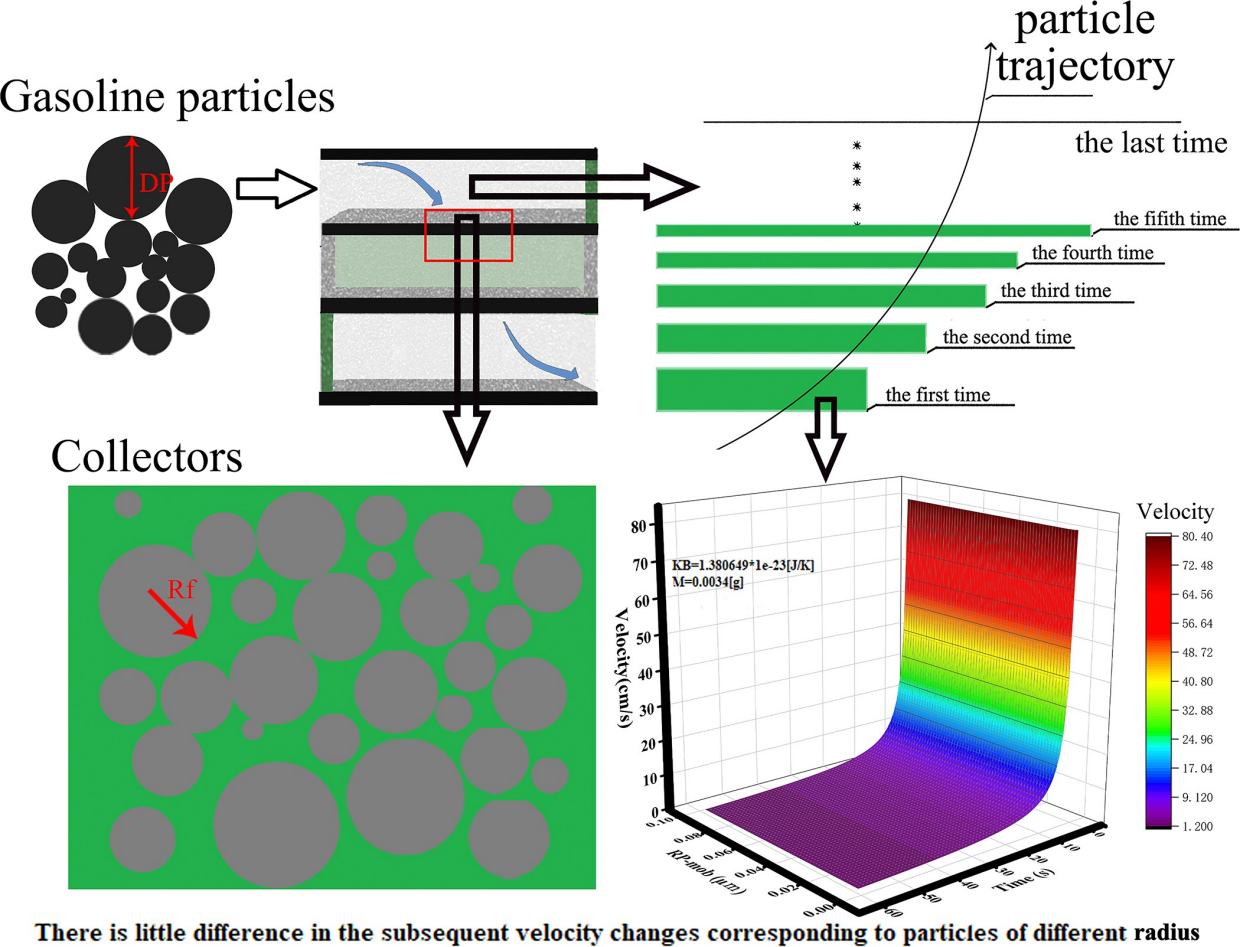
Dynamic trapping process model.

The dynamic trapping process model is shown in Fig 2. As particles enter the trap, they move toward the filter wall due to the influence of airflow in the pore. Under the influence of various mechanisms, the movement speed of particles continuously decreases until they are trapped.

When particles enter the filter layer, their movement speed gradually slows down until they come to a stop. Consequently, the moving distance of particles in unit time undergoes continuous reduction. The change model of the filter layer thickness we have established aligns with this trend. The continuous decrease in the moving distance of particles until they are trapped can be likened to the change in the filter layer, where it continuously decreases until it stabilizes. Based on this observation, the trap can be divided into multiple trapping layers with decreasing thickness.

## Model verification

**Fig 3:**
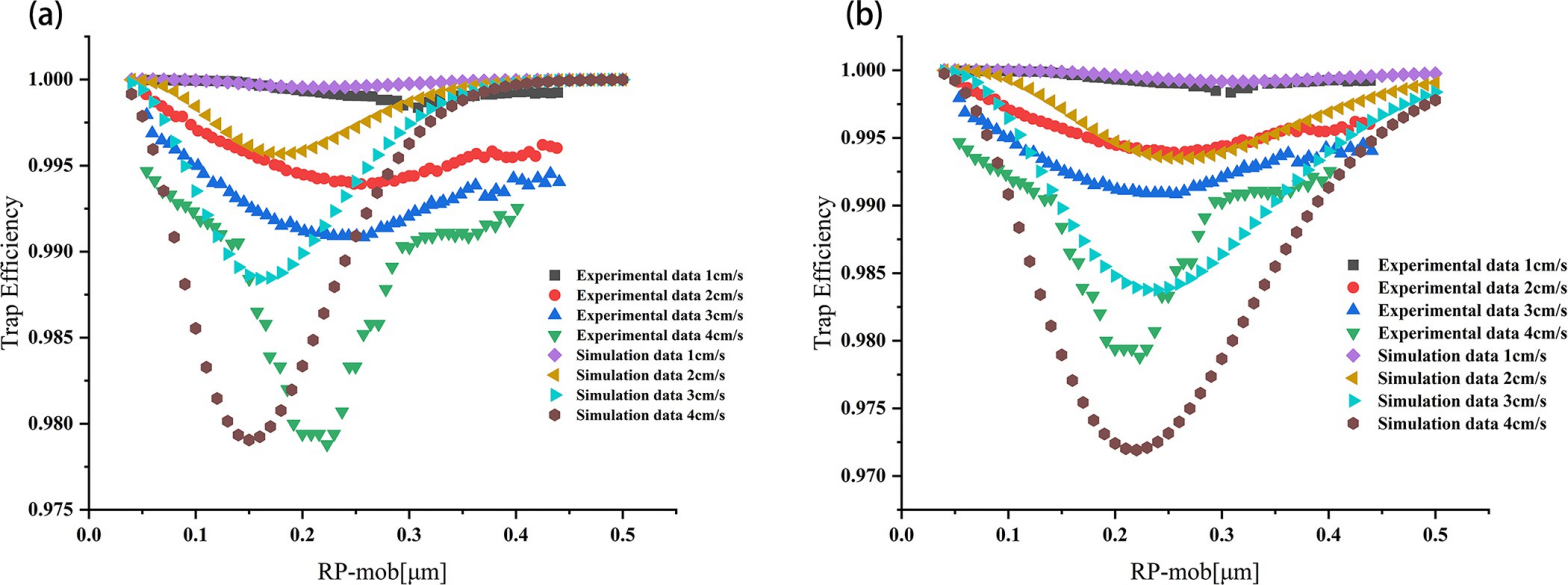
Comparison between simulation results and experimental data of trapping efficiency. (a) Comparison of data before correction; (b) Comparison of data after correction.

The model is verified using the experimental results of trapping efficiency from the literature [34] under different flow rates. The sintered ceramic trap has a filling rate of 0.55, a thickness of 1.65mm, an average pore size of 10.8 μm, and the adopted particle’s fractal dimension is 2.38. Fig 3 compares the simulated trapping efficiency in the particle size range of 50–1000 nm and the experimental data from the literature. To account for the fractal characteristics, the migration equivalent radius is used in the subsequent analysis of particle size.

At flow velocities of 1, 2, 3, and 4 cm/s, the multi-scale non-uniform stratified model shows similarity to the experimental data in predicting smaller fractal aggregates. However, differences arise in predicting the most penetrating particle range and the larger fractal aggregates.

The reason for the difference in the range of the most penetrating particles is mentioned in Balazy’s conclusion on the influence of fractal dimension on trapping efficiency [17]. The range of the most penetrating particle size is affected by the fractal dimension, causing it to shift towards smaller particle sizes with reduced fractal dimension. However, with a smaller fractal dimension, fractal traps are easier to trap compared to spherical traps, resulting in higher trapping efficiency.

The difference in larger fractal aggregates is because their trapping efficiency is independent of airflow velocity, whereas small fractal aggregates can exhibit similar results to spherical particles. For fractal aggregates, the direct interception mechanism (independent of flow velocity) has a weaker effect than the inertial effect.

The particle size range of the most penetrating particle can be accurately predicted by introducing the fractal dimension correction coefficient, where the correction function is ∂ = 3.05691×*Df*^−0.89635^, where ∂ is 1.405. The maximum relative error range is within 0.7%.

## Results and analysis

Based on the trap diameter probability density distribution function, the structural parameters of the MNHF model were set as follows: the average porosity is 0.45, the average pore size is 16 μm, the pore size variance is 2 μm, the fractal dimension of particulate matter is 2, and the primary particle radius is 0.15 μm. The dynamic change process of the characteristic parameters of the wall layer during filtration was analyzed in detail.

### Dynamic changes in porosity and permeability

**Fig 4:**
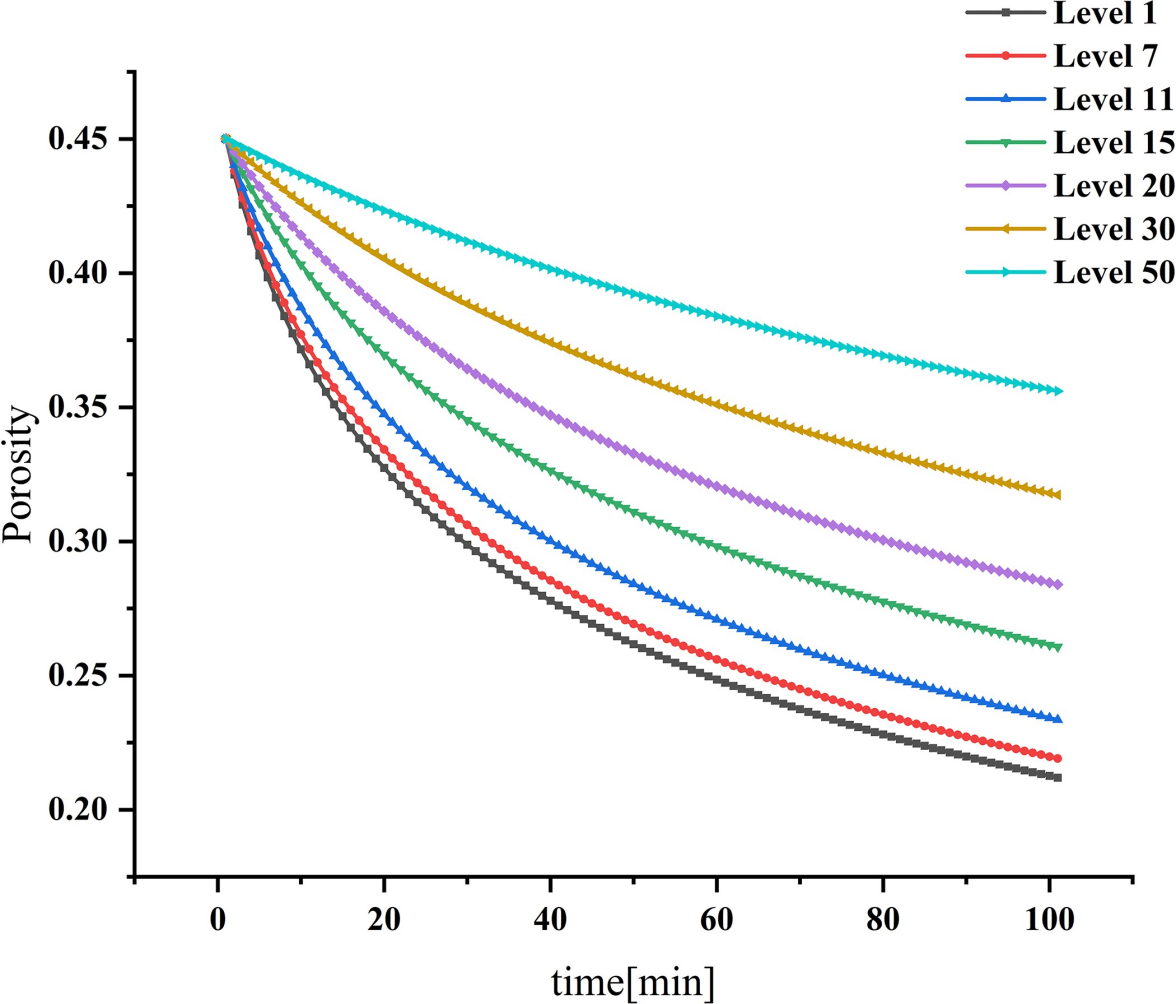
Porosity as a function of time.

**Fig 5:**
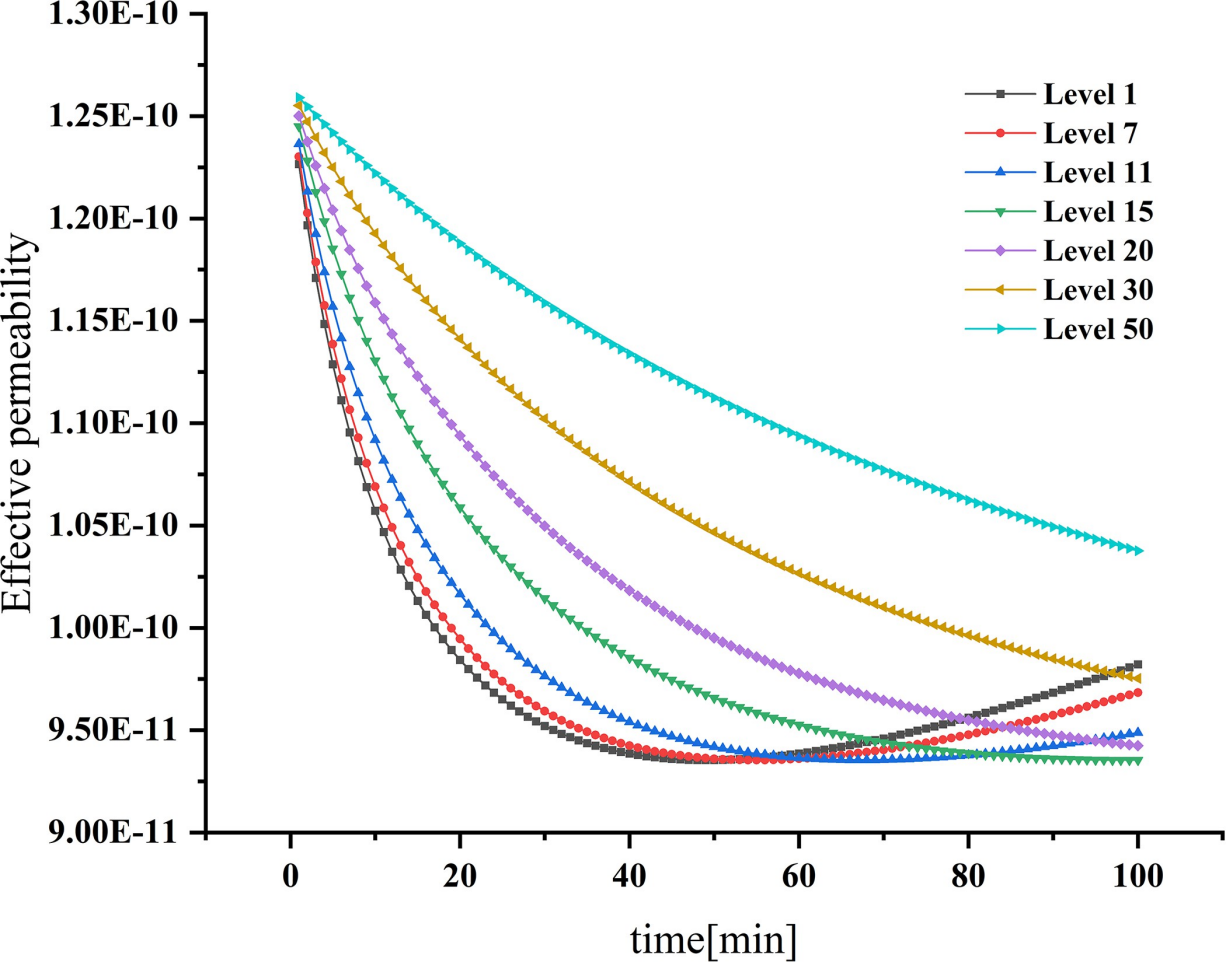
Permeability changes over time.

Figs 4 and 5 show the dynamic changes of porosity and permeability corresponding to the first, seventh, 11th, 15th, 20th, 30th, and 50th layers, respectively, as particles enter the filter wall layer. Ito et al. (Ito et al. 2018) found that the thickness of the plasma wall was low and could not be used as a design optimization scheme for particulate traps. In the MNHF model, the layer thickness decreases with time, and the filter area reduces, leading to a gradual decrease in trapping efficiency layer by layer. The lower the trapping efficiency, the smaller the porosity reduction range. The fastest decreasing porosity is reduced from the initial value of 0.45 to 0.21. As the variation trend of single-layer porosity becomes smaller, the change in trapping efficiency will be affected, resulting in a minor change trend of the filtration rate in the late trapping period, thus affecting the increasing trend of trapped particulate concentration. The lower the trapping efficiency, the smaller the reduction in permeability, and the single-layer permeability shows a decreasing trend with time. The permeability of the first layer decreases to its lowest value of 9.35×10^−11^*m*^2^, at 48 min, while the permeability of the seventh layer and the 11th layer reaches their lowest values at 56 min and 69 min, respectively, representing about 76.6% of the initial value of the pure trap. After reaching the lowest value, the permeability starts to increase, and the increase is more significant for higher initial values of trapping efficiency. The change in permeability primarily affects the pressure drop, which continuously increases with the decrease in permeability, although the increasing trend of pressure drop gradually decreases.

### Dynamic changes in trapping efficiency

**Fig 6:**
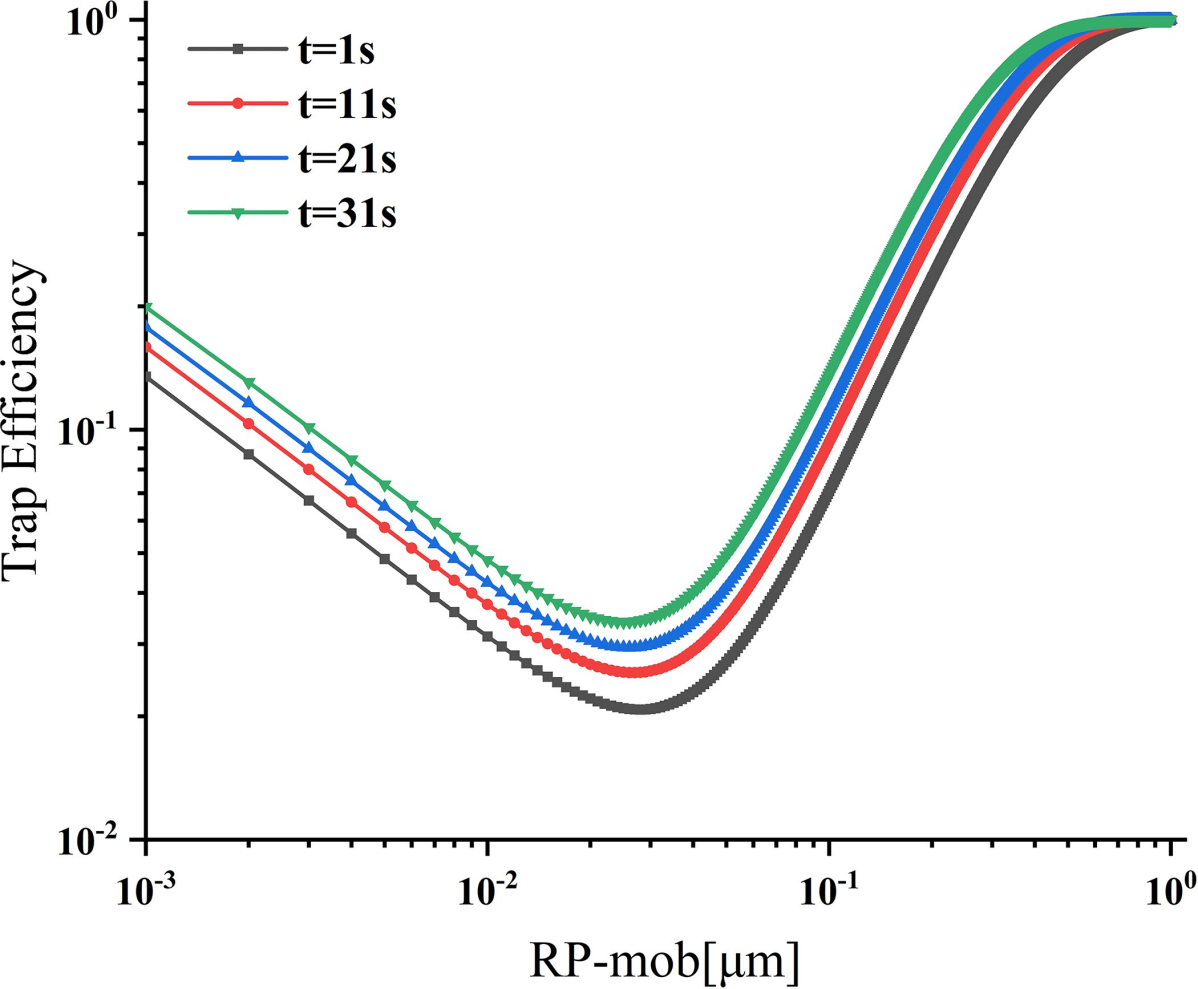
Trapping efficiency of the first layer at different times.

The trapping efficiency of the first layer of the collective at different times is shown in Fig 6. The change in trap diameter over time affects the porosity. The continuous reduction in porosity increases the trapping efficiency. Within the range of particle size variation, there is a trend of high efficiency on both sides and low efficiency in the middle. With the increase in time, the most penetrating particle size shows a decreasing trend. Based on the pore size probability density distribution function, Li et al. [19] found that the size of the effective trap decreases continuously, resulting in a reduction of porosity, and they reached the same conclusion. Since the porosity of the first layer does not vary significantly with time, the collection efficiency of the three data groups (11 s, 21 s, and 31 s) shows a slight increase at four different time nodes: 1 s, 11 s, 21 s, and 31 s. For particle sizes smaller than 0.1 μm, there is a substantial difference in trapping efficiency among different particle sizes. However, when the particle size exceeds 0.1 μm, the growth range of trapping efficiency decreases. This is due to the diminishing change trend of porosity, which is smaller and smaller, which affects the trapping efficiency changes. Consequently, the filtration rate experiences minor changes during the late trapping period, affecting the increased trend of trapped particulate matter concentration. Over time, the single-layer trapping efficiency gradually increases, and as the particle size increases to a certain extent, the increase rate decreases and eventually tends to a fixed value.

**Fig 7:**
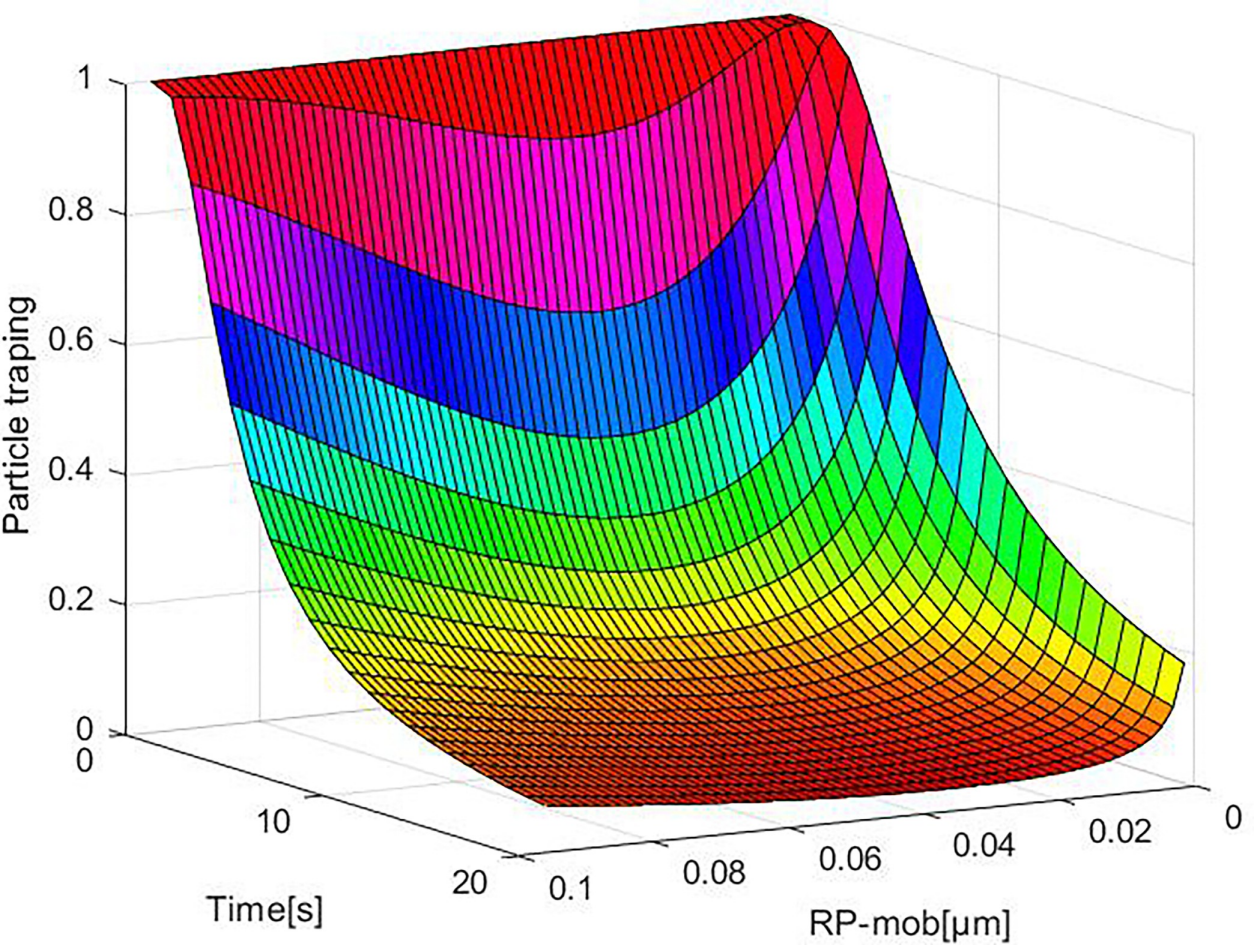
Trapping efficiency of different filter layers and particle migration radius.

The changes in the trapping efficiency of particles with different sizes in various layers are shown in Fig 7. As the thickness of the layers decreases, the difference in trapping efficiency between different particle sizes and different layers also reduces, leading to the tendency of the most penetrating particle sizes tend to fall. The phenomenon occurs because, over the same time, the particles’ moving distance decreases, gradually reducing the trap’s layer thickness. Trapping efficiency is inversely proportional to the number of layers, decreasing as the number of layers increases. Similar to Fig 6, the trapping efficiency changes with different particle sizes exhibit a consistent trend. However, after 11 s of layering, particles in the size range of 0.0016–0.0304 μm show a noticeable variation in trapping efficiency, while particles in other size ranges do not demonstrate a clear trend.

### Pressure drop

**Fig 8:**
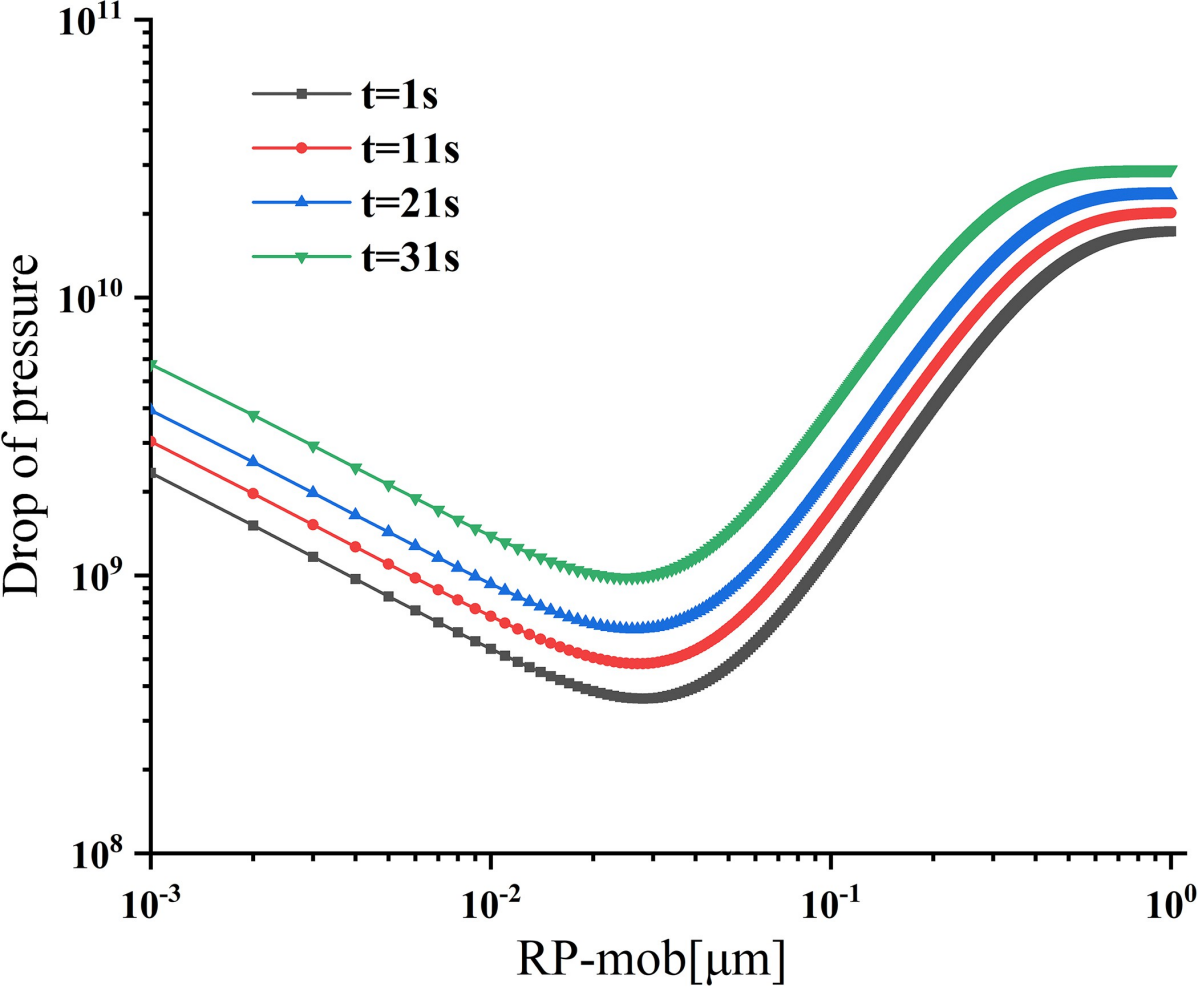
Relation of trap pressure drop at different times in the first layer.

As shown in Fig 8, the pressure drop in the trap changes with time. In the first layer, there is an overall upward trend in pressure drop with time, but the growth rate gradually decreases. This phenomenon is attributed to the impact of permeability changes on the pressure drop. As permeability decreases, the pressure drop increases, but the rate of pressure drop growth diminishes. Serrano et al. [35] found that studying trapping efficiency benefits understanding pressure drop dynamics. The figure shows that the pressure drop difference between different particles remains within a very narrow range when the particle radius exceeds 0.1 μm, which aligns with the trapping efficiency’s change trend.

**Fig 9:**
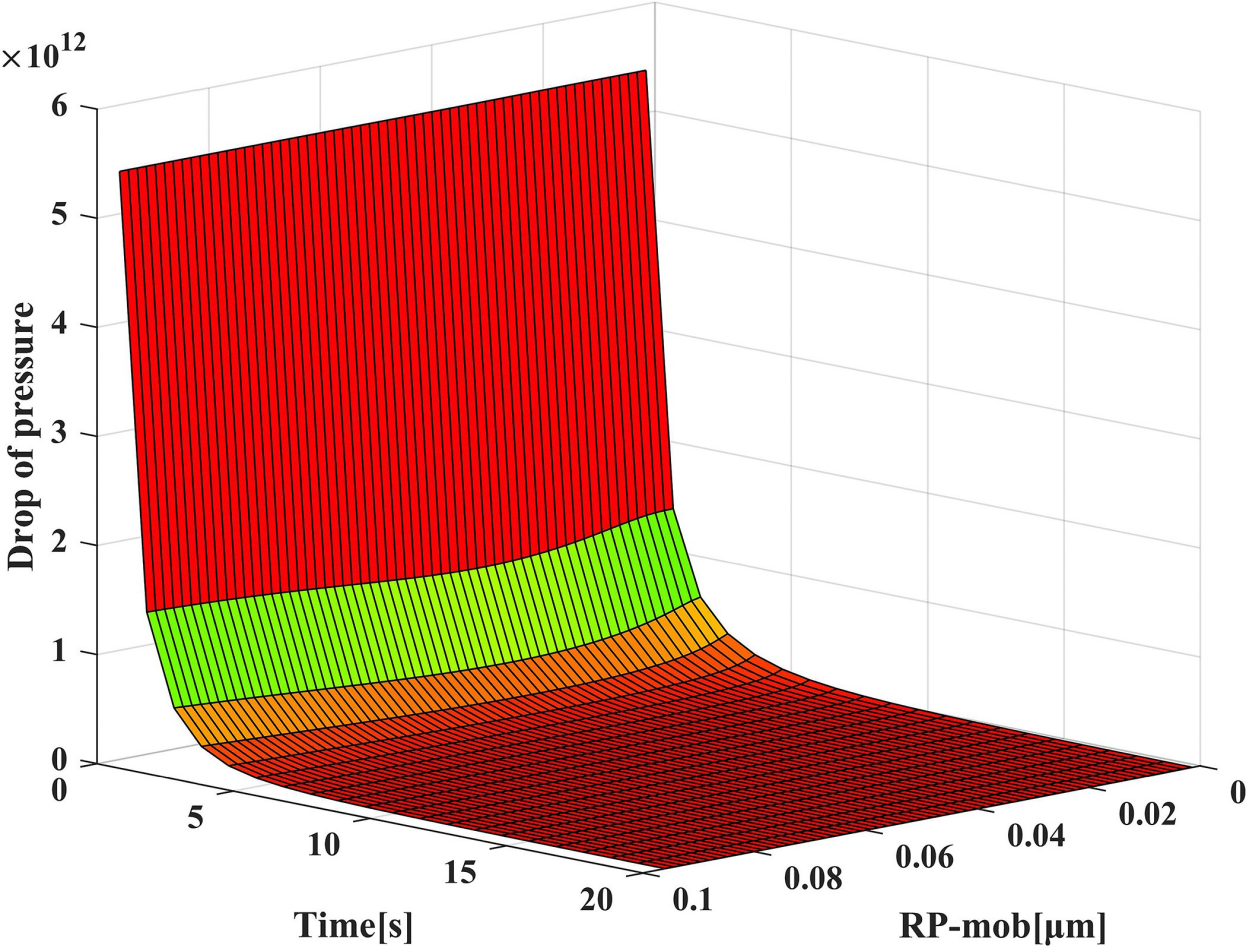
Pressure drop at different filtration layers and migration radius.

The pressure drop of different filtration layers and migration radius are shown in Fig 9. It shows a trend of high values on both sides and low values in the middle, but the difference in pressure drop between different particle sizes becomes relatively small after 3 s. This phenomenon occurs due to the continuous reduction of layer thickness, resulting in a smaller filter area and reducing filter resistance and pressure drop. Within the first 3 s, the pressure drop value varies significantly with the change of thickness, and the maximum change occurs within 0–1 s, accounting for 16.3% of the initial value. As the layer thickness decreases to less than 1.01 mm, the variation range of pressure drop decreases with the influence of layer thickness. Additionally, the increasing range of pressure drop decreases with the increase in particle size.

### Effect of flow velocity on trapping efficiency

**Fig 10:**
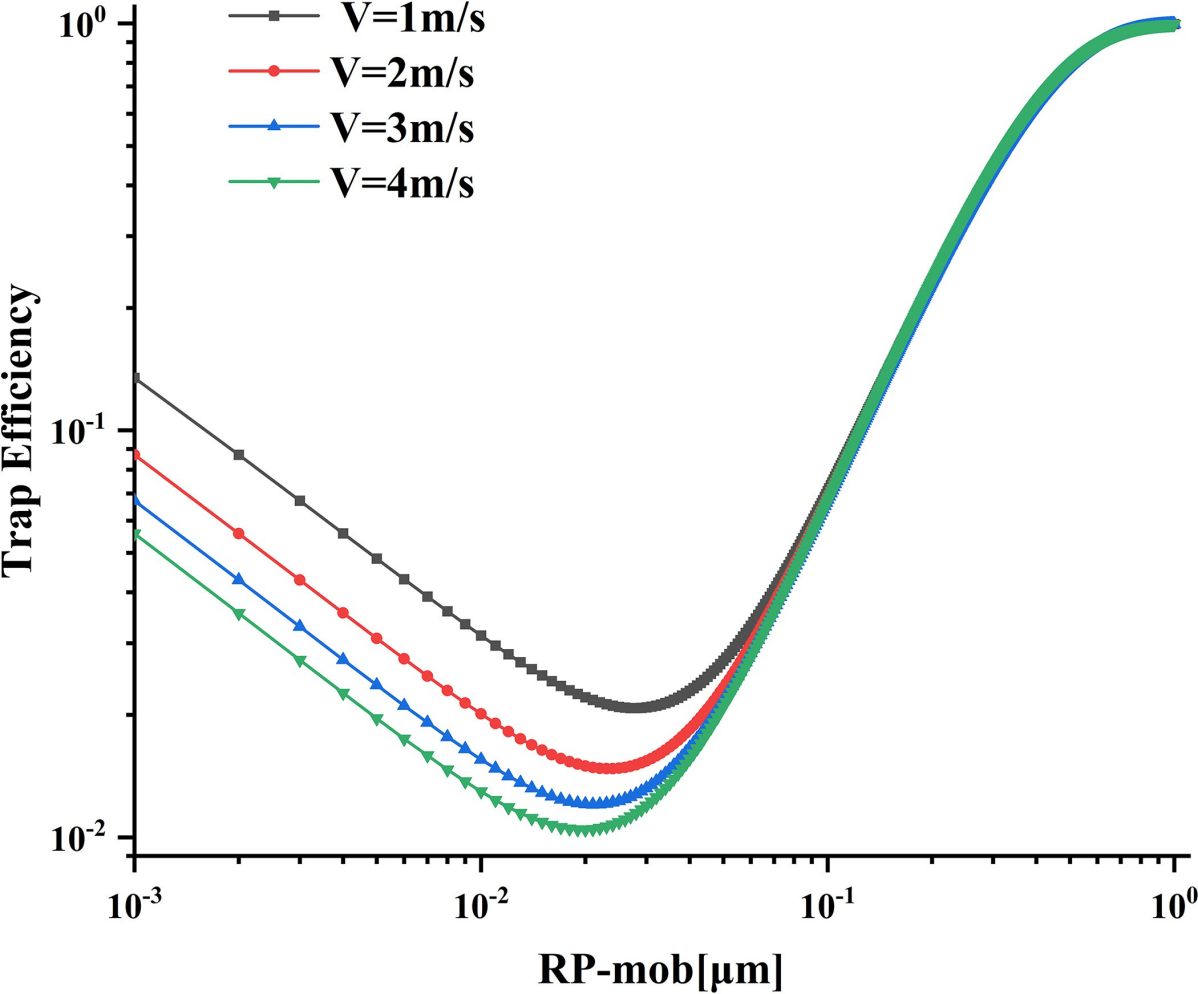
Influence of different flow rates on trapping efficiency.

By simulating different flow rates of 1 m/s, 2 m/s, 3 m/s, and 4 m/s, we studied the influence of flow rate on tapping efficiency. Fig 10 illustrates that when the particle size is less than 0.07 μm, the trapping efficiency exhibits a decreasing trend with increasing airflow velocity. However, for particle sizes larger than 0.07 μm, the trapping efficiency corresponding to different flow rates tends to converge, and with an increase in particle radius, the trapping efficiency shows an upward trend. Additionally, as the flow velocity increases, the most penetrating particles tend to shift toward smaller particle sizes. In Balazy’s research on the effect of velocity on trapping efficiency [24], she points out that the curve has such a changing trend because an increased velocity enhances the penetration of particles, making it more difficult to trap them, thus resulting in this observed change.

### Influence of collective radius on trapping efficiency

**Fig 11:**
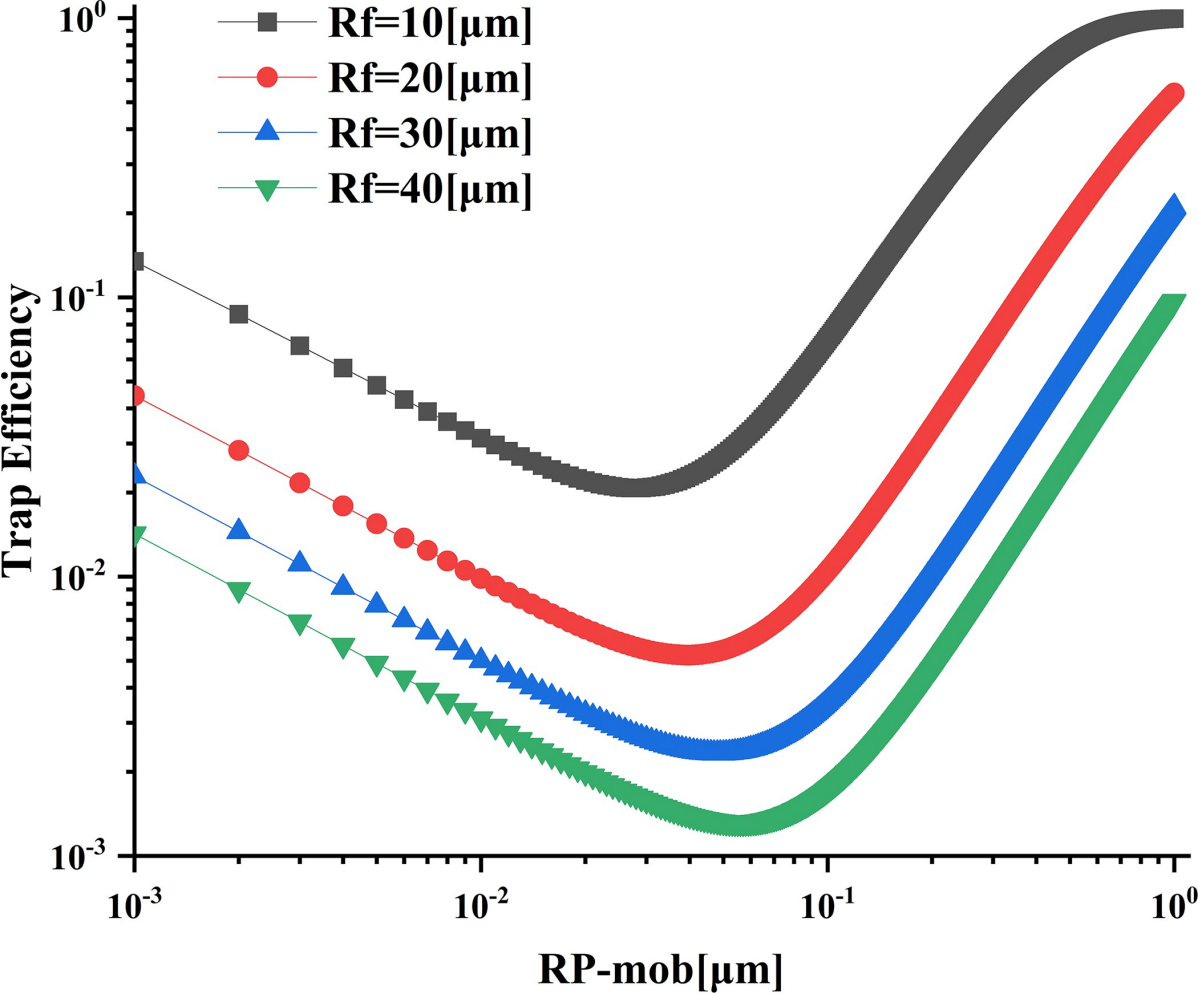
Effects of different fiber radii on trapping efficiency.

As shown in Fig 11, the trapping efficiency exhibits an overall upward trend with an increase in the radius of the trap. This is due to the larger trap radius leading to a reduction in porosity. The most penetrating particle sizes in the range of 10–40 μm correspond to 0.0233, 0.0058, 0.0025, and 0.0015 μm, respectively. Notably, the particle sizes with the highest penetrability tend to move to the larger particle size range. For particle sizes smaller than the most penetrating range, the trapping efficiency shows a decreasing trend as the particle size increases. Conversely, for particle sizes larger than the most penetrating range, the trapping efficiency also shows an upward trend with an increase in the particle radius. Reducing the trap’s radius improves trapping efficiency and narrows the particle size range that reaches the fixed value.

### Effect of particle fractal dimension on trapping efficiency

**Fig 12:**
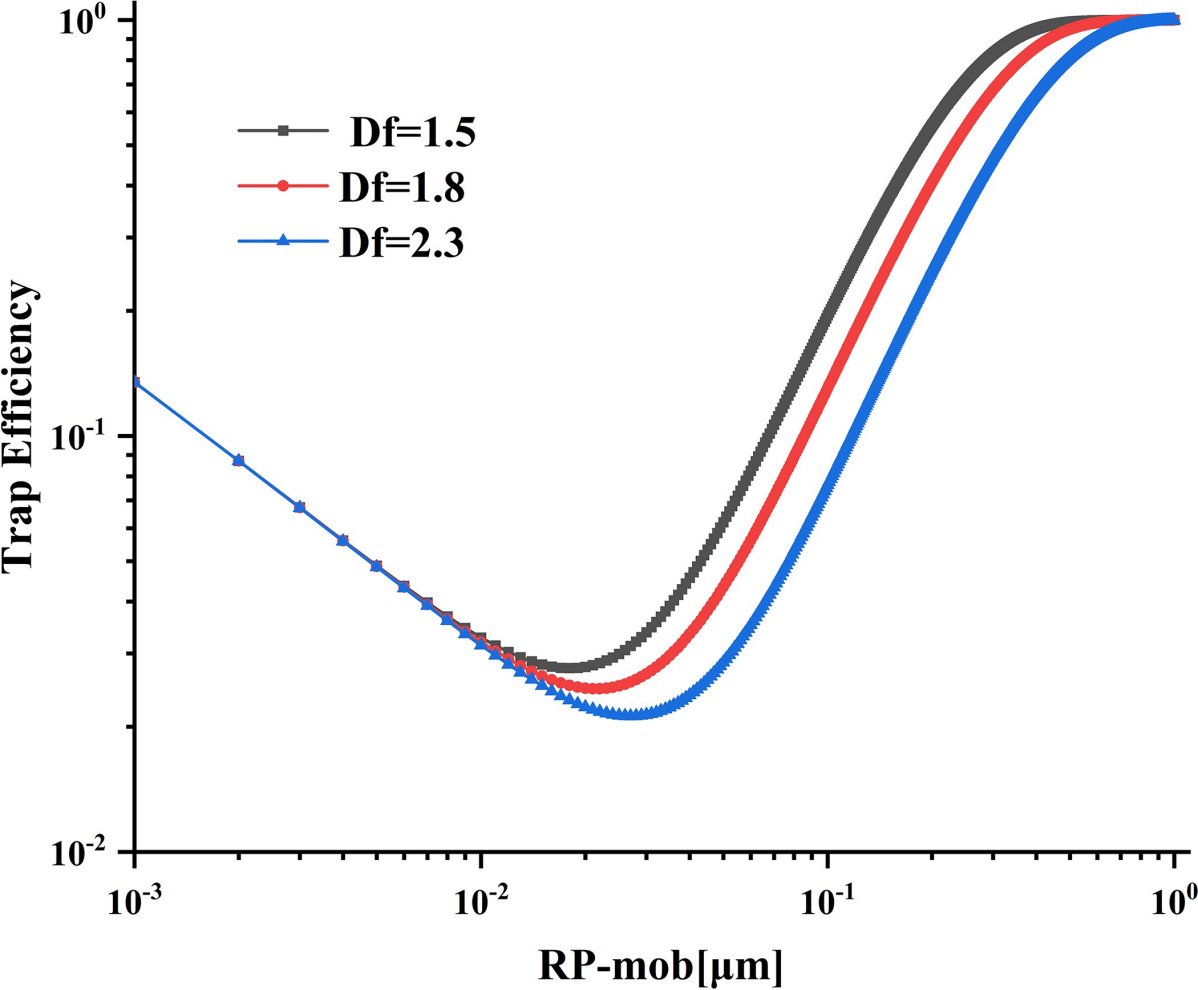
Effects of different fractal dimensions on trapping efficiency.

As shown in Fig 12, when the particle size falls within the range of 0.001–0.01 μm, there is little difference caused by different fractal dimensions, and the trapping efficiency shows a decreasing change process. However, for particle sizes larger than 0.01 μm, the trapping efficiency shows a decreasing trend with higher fractal dimensions, and the most penetrating particles tend to shift towards smaller particle sizes. This behavior can be attributed to the polymer’s interception mechanism. In Balazy’s research on the effect of particulate matter’s fractal dimension on trapping efficiency [24], it is noted that the influence of Brownian diffusion on trapping efficiency decreases with a decrease in fractal dimension. The shell equivalent radius of fractal polymers increases with the decrease of fractal dimension. Fractal polymers’ shell equivalent radius increases as the fractal dimension decreases, leading to an increase in trapping efficiency. Based on the analysis of the influence of fractal dimension on trapping efficiency, it is concluded that the particle size with the highest penetrability ranges from 0.021 to 0.031, and the tapping efficiency ranges from 0.021 to 0.028.

### Effect of wall thickness on trapping efficiency

**Fig 13:**
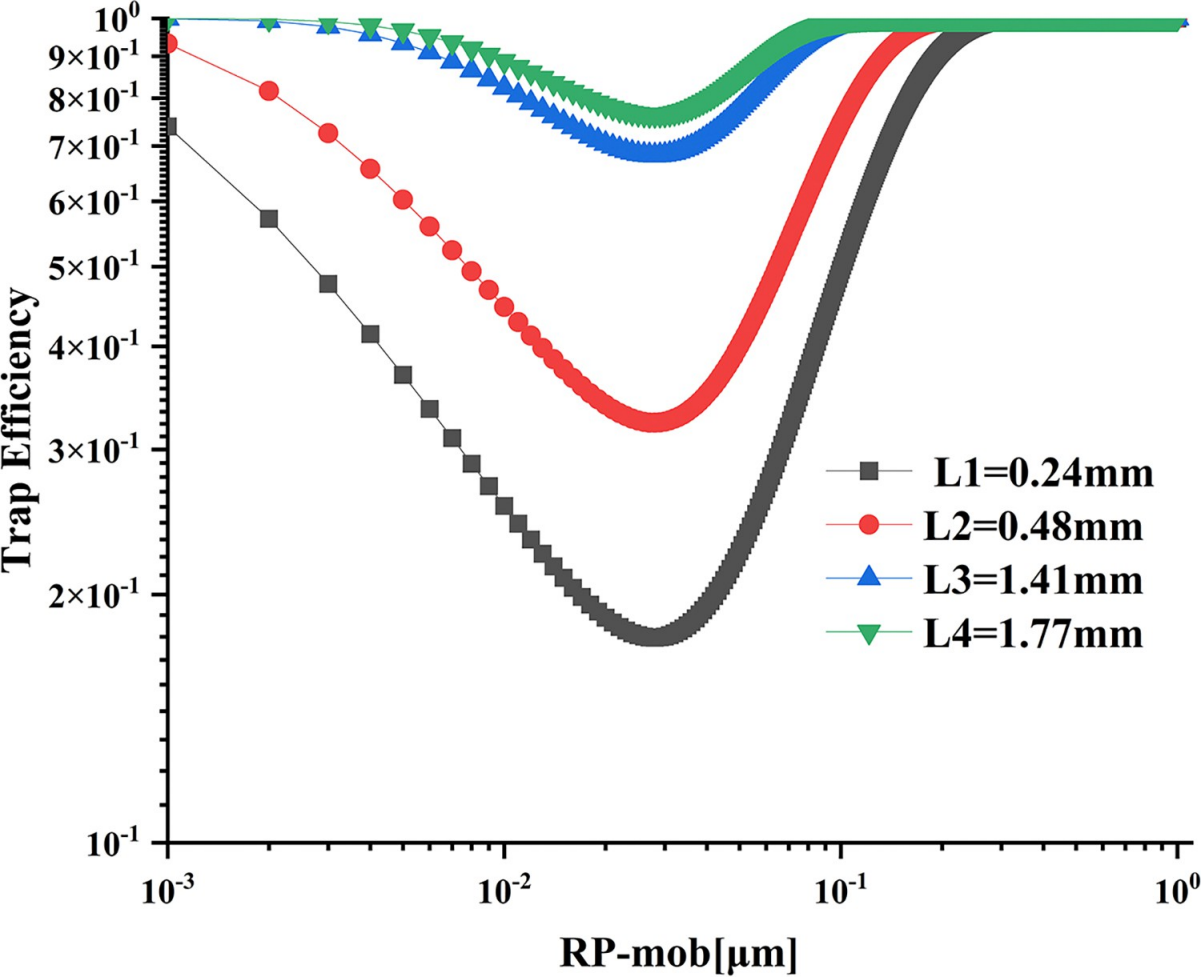
Influence of different wall thicknesses on trapping efficiency.

As shown in Fig 13, an increasing trend in trapping efficiency is observed with the increase in wall thickness. This is attributed to the larger filtration area resulting from the increased wall thickness, which subsequently increases the resistance encountered by particles during passage. For particle sizes within the range smaller than the most penetrating particle size of 0.031 μm, the trapping efficiency decreases correspondingly with an increase in particle size, regardless of the wall thickness. However, for particle sizes larger than 0.031 μm, the changes in trapping efficiency become more pronounced as the particle size increases and the wall thickness decreases. Additionally, a larger wall thickness leads to a smaller particle size at which the trapping efficiency reaches a fixed value. For instance, when the particle size is 0.23 μm, the corresponding trapping efficiencies for different wall thicknesses are 0.071, 0.057, 0.019, and 0.009, respectively, in ascending order.

## Conclusion

The multi-scale non-uniform hierarchical model (MNHF) based on fractal theory is used to accurately analyze the dynamic change of trapping efficiency during the trapping process of GPF. Considering the fractal characteristics of gasoline engine particulate matter, based on the probability density distribution function of the collector diameter, the multi-scale characteristics of the filter wall layer are represented by using the relationship between the collector diameter and the pore diameter. When predicting the dynamic changes of filtration efficiency and pressure drop during the filter trapping process, compared with other dynamic models, by introducing the Brown Dynamics method, the randomness of the movement of particles during the trapping process can be reflected. Then, in the filtration process, by applying the comprehensive change relationship of filling rate, collector diameter and particulate matter mass over time, the dynamic analysis of trapping efficiency in the filtration process is realized.

After the dynamic analysis of filtration efficiency and pressure drop, it is found that the pressure drop and trapping efficiency increase with time when the regeneration mechanism is not considered. The moving distance of particles with different migration radii within the same time decreases, resulting in a gradual reduction in the stratified thickness of the collector. Therefore, the single-layer trapping efficiency decreases with the increase of the number of layers. In the most penetrating particle size range, the decrease in trapping efficiency can be up to 29.34%. When the migration radius is less than 0.07 μm, the trapping efficiency shows a decreasing trend as the air flow velocity increases. When it is greater than 0.07 μm, the trapping efficiency at different flow velocities is almost the same. With the continuous increase of the collector radius and wall layer thickness, the trapping efficiency shows an increasing trend. In the migration radius range of 0.001–0.01 μm, the fractal dimension of particulate matter has little influence on the trapping efficiency. In the migration radius range greater than 0.01 μm, the higher the fractal dimension, the lower the trapping efficiency. In optimizing the performance of the particle catcher, appropriate reductions in flow rate can be achieved by either decreasing the trap radius or increasing the thickness of the wall layer. Additionally, controlling the porosity and permeability within a certain range while considering the balance between pressure drop and trapping efficiency is crucial.

With the accumulation of particles, the actual GPF trapping process can be divided into the following four stages: deep-bed filtration stage, transition stage, filter cake filtration stage, and regeneration stage. The difficulty in predicting trapping performance in the subsequent stages arises from the complex changes in structural parameters. The MNHF model provides a more accurate reflection of trapping performance by considering the non-uniformity of particle movement distances at different times. In the subsequent trapping process, identifying the stage of the trap can be judged by analyzing the moving distance of particles, with a focus on monitoring changes in resistance during the process. Moreover, taking into account the non-uniformity of the actual porosity distribution can improve the overall prediction accuracy of the trap efficiency. In the study of particulate matter in gasoline engines, while the MNHF model’s particle size range is limited when examining trapping performance changes, further improvement can be achieved through a deeper application of fractal theory. This includes considering aspects such as the selection of fractal characteristics of the structure and friction coefficient.
